# Useful Electrocardiographic Signs to Support the Prediction of Favorable Response to Cardiac Resynchronization Therapy

**DOI:** 10.3390/jcdd10100425

**Published:** 2023-10-14

**Authors:** Andras Simon, David Pilecky, Loretta Zsuzsa Kiss, Mate Vamos

**Affiliations:** 1 Department of Cardiology, Szent Imre University Teaching Hospital, 1115 Budapest, Hungary; dr.andras.simon@gmail.com; 2 Gottsegen National Cardiovascular Center, 1096 Budapest, Hungary; pileckyd@gmail.com; 3 Doctoral School of Clinical Medicine, University of Szeged, 6725 Szeged, Hungary; 4 Heart and Vascular Center, Semmelweis University, 1122 Budapest, Hungary; kisslotti@gmail.com; 5 Cardiac Electrophysiology Division, Department of Internal Medicine, University of Szeged, 6725 Szeged, Hungary

**Keywords:** cardiac resynchronization therapy, CRT, ECG, prediction, response, left bundle branch block, LBBB

## Abstract

Cardiac resynchronization therapy (CRT) is a cornerstone therapeutic opportunity for selected patients with heart failure. For optimal patient selection, no other method has been proven to be more effective than the 12-lead ECG, and hence ECG characteristics are extensively researched. The evaluation of particular ECG signs before the implantation may improve selection and, consequently, clinical outcomes. The definition of a true left bundle branch block (LBBB) seems to be the best starting point with which to select patients for CRT. Although there are no universally accepted definitions of LBBB, using the classical LBBB criteria, some ECG parameters are associated with CRT response. In patients with non-true LBBB or non-LBBB, further ECG predictors of response and non-response could be analyzed, such as QRS fractionation, signs of residual left bundle branch conduction, S-waves in V6, intrinsicoid deflection, or non-invasive estimates of Q-LV which are described in newer publications. The most important and recent study results of the topic are summarized and discussed in this current review.

## 1. Introduction

Biventricular pacing significantly decreases QRS duration and consequently improves cardiac performance [1]. Several major randomized studies [2,3,4,5,6,7]—with the widening of the QRS as the main inclusion criteria—have shown that CRT is beneficial in patients with heart failure with reduced systolic function. Alongside pharmacologic treatment, it is proven to be an effective tool to reverse ventricular remodeling and reduce the rate of hospitalization and improve survival.

While the clinical indication for CRT has in essence remained stable, the criteria relating to ECG parameters have changed over time. Previously, it was mainly the QRS duration that determined which—otherwise optimally managed, but still symptomatic—patients with persistently impaired left ventricular systolic function should receive CRT. Previous guidelines [8,9] have determined this value as ≥120 ms. In case of a narrow QRS, CRT was not only proven to be less useful, but it seemed to be disadvantageous as it increased mortality even if left ventricular dyssynchrony was evident by echocardiography [10,11,12]. Meanwhile, accumulating evidence showed that in one third of the patients receiving CRT, no improvement in symptoms and/or in left ventricular ejection fraction could be observed [13,14]. 

Additional data also showed that for patients with symptomatic heart failure, the wider the QRS is, the greater the benefit can be obtained from CRT with regard to hospitalization and mortality [3]. Furthermore, a meta-analysis of randomized trials drew attention to the finding that patients with moderate QRS prolongation (i.e., 120–149 ms) might not benefit from CRT with respect to adverse clinical events, in contrast to patients having a QRS duration of more than 150 ms [15]. 

Beyond the matter of QRS duration, morphological criteria have also been added to the guidelines. Patients with a left bundle branch block (LBBB) pattern demonstrated more benefit than those with right bundle branch block (RBBB) or non-specific intraventricular conduction delay (IVCD) patterns [16,17,18]. Patients with the combination of a normal PR interval and non-LBBB morphology may even have a higher mortality risk after CRT [19]. Currently, only symptomatic patients with LBBB and a QRS duration of at least 150 ms in their native ECG have a class IA indication for CRT. Recommendations are weaker for patients with an LBBB pattern and a QRS duration of >130 ms and patients with a non-LBBB pattern and a QRS duration of >150 ms, and questionable for patients with a non-LBBB pattern and a QRS duration of 130–149 ms [20,21].

In order to more precisely distinguish patients for whom the treatment is useful from those who presumably do not benefit from cardiac resynchronization, electrocardiographic signs seem to be the most valuable tools, and thus have been extensively researched. The current descriptive review aims to summarize the most relevant and recent studies of this topic for daily clinical practice. Notably, most of the referred CRT trials applied echocardiographic and/or soft clinical endpoints (i.e., improvement of NYHA class, change in NTproBNP), but in some of them, the CRT response was rather based on hard clinical outcomes (such as lack of hospitalization or mortality).

## 2. The problem of Defining the “True” Left Bundle Branch Block

LBBB is defined differently by the WHO, in the American and European guidelines, and also in major clinical CRT studies, like MADIT-CRT and REVERSE [18,21,22,23,24] (Table 1). Patients with wide QRS who do not meet left bundle branch block definitions and who are thus classified as non-LBBB patients receive different recommendations for CRT. If strict criteria are applied during patient selection, potential responder patients may be deprived of receiving CRT. On the other hand, if less strict criteria are used, patients who may not have any benefit may receive CRT and incur unwanted consequences, such as costs or possible complications. 

According to theoretical considerations, CRT is less useful if the failure of the intraventricular electrical impulse propagation is distal to the specialized conducting system and the delay is in the ‘myocyte to myocyte’ phase. However, the actual site of the block in the left bundle cannot be determined with certainty based on the surface 12-lead ECG.

**Figure 1:**
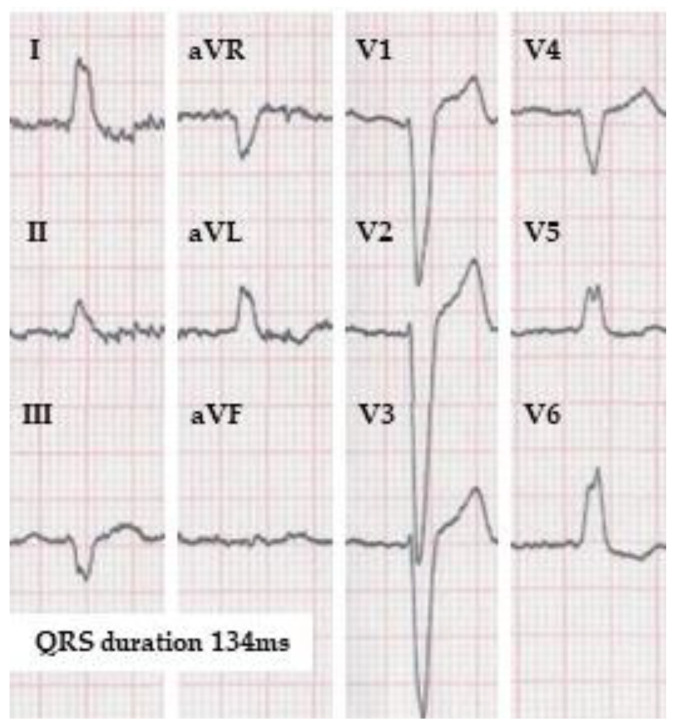
ECG of a patient fulfilling the strict left bundle branch block definition according to Strauss et al. in case it belongs to a female, but not to a male. The QRS duration is 134 ms (by the automatic measurement). Strauss et al. propose QRS duration limit for strict LBBB as 130 ms for women and 140 ms for men. (10 mm/mV, 25 mm/s).

Strauss et al. [23] pointed out that 30% of the patients diagnosed with LBBB actually have the combination of left anterior fascicular block and left ventricular hypertrophy. For this reason, they suggested raising the conventional QRS duration limit of 130 ms. This is because in a real LBBB it takes at least 40 ms for the impulse to get from the right ventricular endocardium to the left ventricular endocardium. From there on, an additional 50 ms is required to reach the posterolateral wall, and an additional 50 ms to radially spread and activate the posterolateral wall. Regarding the individual (e.g., gender) differences in ventricular wall thickness, this altogether lasts for 130–140 ms at the lowest estimate in adults (Figure 1). Moreover, they considered the presence of mid QRS notching and/or slurring in two or more contiguous leads (V1, V2, V5, V6, I, and aVL) as a very important sign. The first notching represents the depolarization wavefront reaching the endocardium of the left ventricle, and the second reaching the epicardium of the posterolateral wall. Padanilam et al. [25] also proposed stricter criteria for a real complete LBBB. They suggested excluding patients who have a septal left-to-right activation. According to their findings, an initial R wave ≥1 mm in lead V1 is a sign of a septal left-to-right activation and represents an intact conduction pathway through the left septal branches.

Patients having a real complete LBBB after suffering a myocardial infarct, may subsequently have a pathological Q-wave in the lateral leads or a high amplitude R-wave evolving septally. According to some LBBB definitions, these patients should be classified into the nonspecific intraventricular conduction disturbance group, though the conduction disturbance of the left bundle would remain. One might presume that applying CRT is suboptimal in these cases, as possibly no significant hemodynamic effect can be expected if we pace in the area of a non-excitable fibrotic scar. Similarly, if pacing affects a late activating territory outside the scar but without enough remaining contractile tissue, any relevant reverse remodeling is questionable. These scenarios might explain why non-ischemic etiology predicts better CRT response than ischemic etiology does [26,27].

The uncertainty related to the definition of LBBB is well demonstrated in the study of van Stipdonk et al., where the preprocedural ECGs of 1492 patients who underwent CRT implantation were analyzed [28]. They found that only 13.8% of the patients could be classified as LBBB by all of the LBBB definitions used in the American and European guidelines, in the MADIT-CRT study, and according to Strauss et al. Moreover, they have found that from the eleven individual ECG characteristics of the different LBBB definitions, only three were independently associated with clinical outcomes. These signs were QS or rS in lead V1; notching or slurring in lead V5, V6, I or aVL; and the absence of a Q wave in leads V5, V6, I and aVL. For note, the primary endpoint of the study of van Stipdonk et al. was a left ventricular assist device implantation, cardiac transplantation, or all-cause mortality, and not echocardiographic and/or clinical/laboratory endpoints.

It is notable that, whichever definition was used for a study, LBBB was always associated with a better outcome in resynchronization therapy when compared to non-LBBB. Furthermore, most of the studies found that patients who had “true LBBB”, according to the stricter definition of LBBB, had a greater improvement in left ventricular function compared to other patients selected for CRT [29,30,31,32]. The ECG signs detectable by the human eye on the conventional 12-lead ECG that are used to predict response to CRT in cases of true LBBB are summarized in the Graphical Abstract and Table 2. Supplementary Table S1 summarizes the specificity and sensitivity data if they were available in the original papers. It is worth noting that a direct comparison of these parameters could be misleading since they arise from different patients’ cohorts.

## 3. ECG Signs Predicting CRT Response beyond the Classical LBBB Markers

Based on the evidence described above, the definition of a true LBBB seems to be the best starting point with which to select patients for CRT. In patients with non-true LBBB or non-LBBB, further ECG predictors of response and non-response could be analyzed. They are also summarized in the Graphical Abstract, and Table 2 and Table S1.

### 3.1. QRS Fractionation

Significant effort has been made to estimate the extent of myocardial fibrosis or scarring and their association with CRT response. The short-duration fractionation caused by left ventricular dyssynchrony is typically located at the mid-QRS. More than two notches of the R wave or in the nadir of the S wave is unexpected in left ventricular dyssynchrony. The more leads with fragmented QRS, the worse the response was to CRT [33]. In patients with LBBB and a non-ischemic etiology of heart failure, a shorter time (<32.5 ms) from the QRS onset to the beginning of the QRS fragmentation, and a longer fractionation duration, also predicted CRT non-response [34]. On the contrary, the study of Rickard et al. [35] did not support this observation. According to their findings, QRS fragmentation was not associated with a poor response to CRT.

In a small study amongst patients with LBBB, the longer time (≥45 ms) between the peak of the R to the nadir of the S wave in V1 (representing the activation of two opposite regions of the heart) predicted a better response to CRT [36]. 

### 3.2. Signs of Residual Left-Bundle Branch Conduction

The q waves in lateral leads can indicate either a residual LBB conduction or apical myocardial infarction or cardiac fibrosis, each resulting in a significantly lower rate of CRT response in patients with LBBB [37]. Similarly, an R wave in V1 ≥1 mm may either signify a residual LBB conduction, or a previous posterior wall infarction. The absence of an R wave in V1 (≥1 mm) also predicts a greater response to CRT [32].

### 3.3. S-Waves

An S wave in V6 has a high predictive value to identify patients with complete LBBB who may have poor CRT response [38]. The reason for the presence of an S wave in V6 in complete LBBB is not yet unequivocally explained. A simultaneous delay in the activation of the right ventricle (for example because of a right ventricular pressure overload and enlargement), or posterosuperiorly pointing to late left ventricular activation because of biventricular enlargement [39], or an apical location of a branch of the left posterior fascicle [40], are suspected. Poposka et al. [41] also found that a larger R/S amplitude ratio in V6 is a predictor of CRT response in both LBBB and non-LBBB patients. Another similar marker which may predict CRT response in LBBB is the lead one ratio (LOR) [42]. This is calculated by dividing the measured maximum positive and the maximum negative amplitudes of the QRS complex in lead I. A LOR <12 signifies poor CRT response.

### 3.4. Axis Deviation

The presence of a left or right axis deviation showed controversial results in different publications; therefore, a clear conclusion regarding the association between CRT response and axis deviation cannot be drawn [43,44,45]. Nonetheless, patients with left axis deviation tended to have a shorter left ventricular activation delay, a more extensive apical and anteroseptal scar volume, and more pronounced hypertrophy, reflecting an underlying more advanced structural disease compared to patients with a normal axis.

### 3.5. Universal QRS Signs of Delayed Left Ventricular Activation

With the help of some newly recognized parameters of the 12-lead ECG, a significant portion of patients could be identified as having a delayed left ventricular activation but categorized as non-LBBB according to the strict LBBB criteria; consequently, they may be excluded from CRT. 

The intrinsicoid deflection (ID), which is the time from the earliest onset of the QRS to the point where the maximum deflection towards the baseline starts, represents the time when the electrical activation reaches the cardiac area beneath a particular lead. It seems reasonable that the prolongation of the ID in lateral leads predicts response to CRT. Del-Carpio Munoz et al. [46] found that the ID in lateral leads is a better predictor of CRT response (with a cut-off point of 110 ms in lead I, 130 ms in lead aVL, ID in lead I/QRS duration >0.69, and a difference of >90 ms between ID in lead I and V1), than QRS duration itself.

Vereckei et al. created two formulas to estimate the left ventricular intra- and interventricular dyssynchrony [47]. The intraventricular dyssynchrony is characterized by the difference of the ID times of the lateral wall (aVL) and the inferior wall (aVF) divided by the total QRS duration: [aVLID − aVFID]/QRSd (%). Interventricular dyssynchrony is calculated as the difference of the intrinsicoid deflection of the left (V5) and right (V1) ventricle divided by the total QRS duration [V5ID − V1ID]/QRSd (%). According to their findings, a value greater than 25% in either case was predictive for CRT response, predominantly in the IVCD group. 

**Figure 2:**
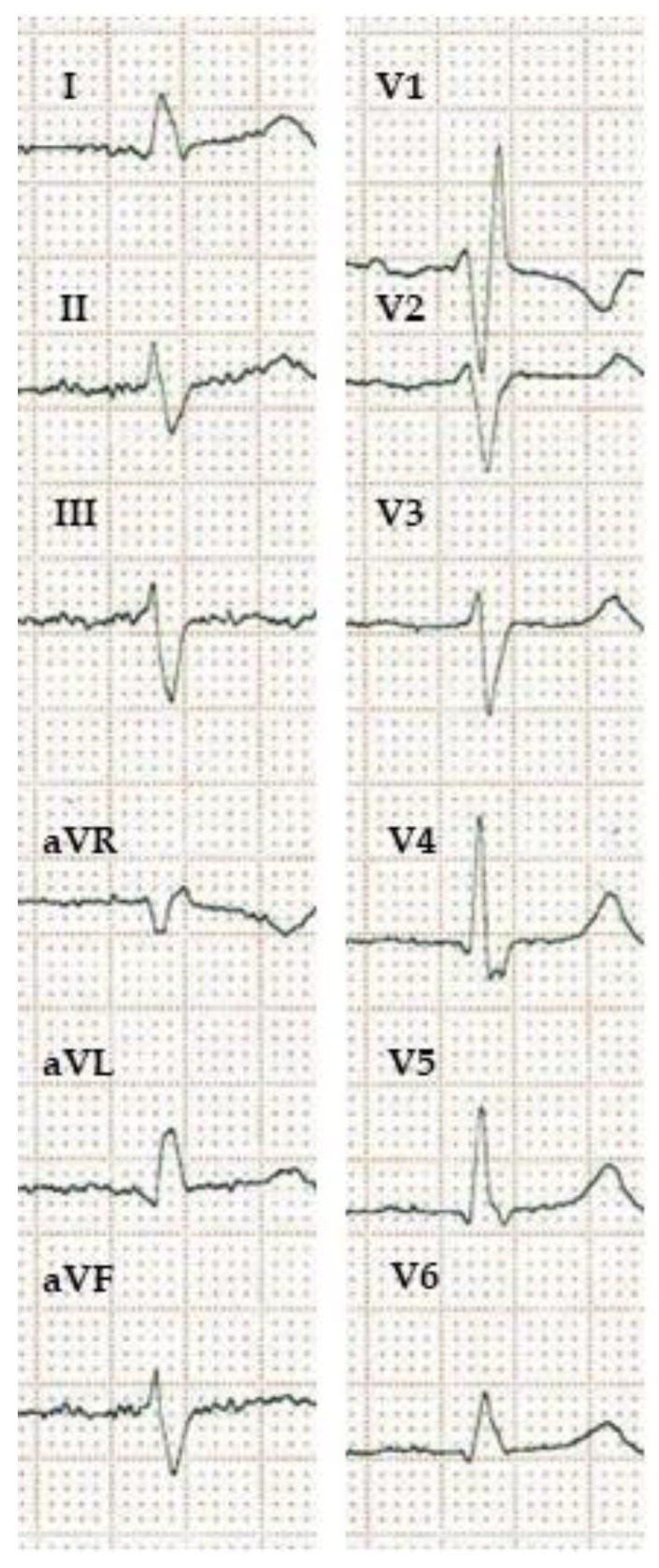
ECG showing masquerading bundle branch block. Masquerading bundle branch block refers to an intraventricular conduction disturbance signified by a right bundle branch block pattern in the precordial leads with the absence of a wide negative terminal deflection in the lateral limb and/or chest leads and a left bundle branch block-like pattern in the lateral leads. It indicates both right and left ventricular activation delay and usually poor prognosis. Patients with masquerading bundle branch block may have a good response to CRT. (10 mm/mV, 25 mm/s).

The Q-LV interval is the time measured from the onset of the QRS (representing the activation of the septum) on the 12-lead ECG, to the first large peak of the left ventricular electrogram obtained from the left ventricular pacing lead. This is invasively acquired data, which helps to select the latest activating ventricular region from the potentially reachable pacing sites. A longer Q-LV interval is strongly associated with better clinical response [48,49]. The comparison of Q-LV with non-invasively obtainable 12-lead ECG parameters revealed that mid-QRS notching and/or slurring, even in one lateral lead, a QRS duration greater than 150 ms, and an ID longer than 60 ms in V6, are associated with a prolonged Q-LV in patients who did not meet the “true LBBB” criteria proposed by Strauss et al. In their study, they also found that patients with atypical RBBB (RBBB morphology in the chest leads, but with the absence of a wide negative terminal deflection in the lateral limb leads) had a long (>110 ms) Q-LV as well [50]. This kind of “atypical RBBB” that resembles masquerading bundle branch block [51]—first described by Richman and Wolff in 1954 [52]—is well known to be associated with poor prognosis [53] (Figure 2). This characteristic helps to identify patients with RBBB who not only have a delayed right ventricular activation, but also a delay of activation of the free left ventricle wall [54]. These patients may also show a good response to CRT [55].

On the basis of the observation of Hara et al. [56] that the QR interval measured from the QRS onset to the R wave offset in the left chest leads correlates with the degree of left ventricular activation delay irrespective of the QRS duration and morphology, another index number was evaluated in patients with non-LBBB. The QR-max index [57] eliminates the axis dependency of the QR interval method. The QR-max index is the longest interval measured in the limb leads from the QRS onset to the R wave offset, which is defined as the crossing point of the descending limb of the R wave and the baseline. With a cut-off value of 120 ms, this better identifies CRT responders among non-LBBB patients than a QRS duration greater than 150 ms according to the guidelines.

### 3.6. The Prolongation of the PR Interval

The literature regarding the association between a baseline PR interval and CRT response is controversial. In general, patients with a prolonged PR interval are known to have worse outcomes compared to those with a normal PR [58]. Kutyifa et al. have shown that patients with non-LBBB and a prolonged PR had better outcomes than patients with non-LBBB and a normal PR [19]. Similarly, Lin et al. [59] found that a prolonged PR interval was associated with a better outcome compared to a normal PR in both LBBB and non-LBBB patients. In contrast, according to the recent study of Atwater et al. [60], when compared to a normal PR, a prolonged PR (>200 ms) in LBBB is predictive of a higher rate of adverse clinical endpoints after CRT implantation. A prolonged PR with wide QRS may signify an underlying diffuse myocardial disease affecting the whole heart or the conduction system. This can mean a wide spectrum of diseases. Among them, there are some, such as cardiac fibrosis, which cannot be influenced by CRT. 

## 4. Limitations

One of the main limitations of reviews or meta-analyses of CRT is the lack of a unique definition of CRT response. This also applies to the current review. In particular, the varying definitions applied to CRT response in the referred articles should be highlighted (i.e., most of them applied echocardiographic and/or soft clinical endpoints, but some of them used hard clinical outcomes, such as lack of hospitalization or mortality). Moreover, we acknowledge that response to CRT depends on multiple parameters, such as the position of the left ventricular lead, biventricular pacing percentage, and medical therapy, which were not evaluated in the current paper.

It is also important to acknowledge that some ECG patterns, which have a role in classifying patients into the LBBB or nonspecific intraventricular conduction disturbance groups, might be significantly influenced by technical and anatomical factors, or are potential victims of subjectivity. For example, there is often variability in the placement of the precordial leads, and even if they are positioned accurately, the actual position of the heart within the chest may vary in the same patient in different conditions. Machine sensitivity and settings are also important influencing factors, which determine the visualization of notching and slurring, and which are also voltage-dependent parameters. Furthermore, there is also a lack of consensus in the measurement of the QRS duration and in the QRS limits [61]. All these factors influence intra- and inter-observer variability, which cannot be ignored; among expert clinicians, one out of ten and one out of five may have a different diagnosis classifying LBBB, respectively [62]. 

## 5. Future Directions

Though the aim of this article was to collect and present patterns detectable for the human eye on the conventional 12-lead ECG to predict a favorable response to CRT (Table 2, Graphical Abstract), it must be acknowledged that in the future, computerized techniques will prevail. The prediction of CRT outcomes might go beyond traditional paper-based ECG evaluations and human perception. 

The 3-dimensional (3D) QRS area measurement tools may help to identify responders more objectively for an electrical therapy [63,64,65]. It can be calculated from the 12-lead ECG as the sum of the area under the vectorcardiographic X, Y, and Z leads’ ventricular deflection curves, and seem to be a better predictor of CRT response than the traditional markers appearing in the current guidelines, such as the QRS duration and QRS morphology. The rationale behind this approach is that large electrical activation wavefronts which appear at different times do not extinguish each other. A longer time of delay and a larger excitable electrically active cardiac muscle mass result in a larger 3D QRS area. In contrast, a lesser degree of dyssynchrony and a larger presence of non-conducting tissue (like any scar of different etiology) both result in a smaller QRS area and are known predictors of non-response. Although the automatized QRS area calculation can eliminate some subjective components in the process of CRT response prediction, according to the findings of Nguyên et al. [66], even the QRS area and other voltage-dependent parameters like QRS amplitude, notching and slurring, RS patterns, and ID-time are significantly influenced by the position of the heart. 

Emerging new computer-aided technologies that extract information from the surface ECG parameters, for example high-frequency and ultra-high-frequency 14-lead electrocardiogram techniques, are promising [67].

Machine learning may also be used to accurately identify CRT responder patients before the procedure and adjust the indications for CRT implantation. For example, a recent machine learning study using 12-lead ECG waveforms not only validated the QRS area as a predictor of CRT response, but objectively identified patients having favorable outcomes without the conventional class I ECG indications for CRT, and also those with LBBB, who had poorer outcomes [68].

Evolving new implantation techniques should be also mentioned, like conduction system pacing, that might eliminate these issues in the future, and we will have greater freedom selecting the pacing sites so targeting can be more precise [69].

## 6. Conclusions

Until now, no other modality than electrocardiography has been proven to be better in predicting the clinical response to CRT. The definition of a true LBBB seems to be the best starting point with which to select patients for CRT. From the classical LBBB criteria, the following three seem to be the most important ones: QS or rS in lead V1; notching or slurring in lead V5, V6, I or aVL; and absence of a q-wave in leads V5, V6, I and aVL. It should also be noted that when stricter definitions of LBBB are used, the better predictions for CRT response is expected. 

In patients with non-true LBBB or non-LBBB, further ECG predictors of response and non-response could be analyzed, like QRS fractionation, signs of residual left-bundle branch conduction, S-waves in V6, intrinsicoid deflection, or non-invasive estimates of Q-LV; these predictors might also add some information beyond the classical LBBB markers for predicting CRT response. However, important limitations should be acknowledged. Signs that can be read from the 12-lead ECG can be hard to memorize due to their complexity and diverseness, and their reproducible perceptivity is influenced by technical and human factors. Moreover, the lack of universal definitions has an impact on the applicability of even simple criteria of current CRT guidelines.

Further research on ECG signs predicting CRT response is still important, especially in patients with non-LBBB patterns. Computer-aided automation, machine learning or new implantation techniques will play an emerging role in extending or limiting conventional CRT indications.

## Figures and Tables

**Table 1:** Criteria of left bundle branch block according to the WHO, the AHA/ACCF/HRS, the ESC, the MADIT and REVERSE trials, and Strauss et al.

	WHO [24]	AHA/ACCF/HRS [20]	ESC [21]	MADIT-CRT [18]	REVERSE [22]	Strauss et al. [23]
QRS duration (ms)	120	120	120	130	120	female: 130 male: 140
rS or QS in V1	+	+	+	+	+	+
Transition zone in the precordial leads is displaced to the left	+	-	-	-	-	-
Broad R in I, aVL, V5-6	+	+	+	+	+	-
Notched/slurred R in I, aVL, V5-6	+	+	+	+	-	+
Mid-QRS notching or slurring in at least 2 of leads V1, V2, V5, V6, I, and aVL	-	-	+	-	-	+
RS pattern allowed in V5-6	+	+	-	+	+	+
No q allowed in V5-6	+	+	+	+	+	-
No q allowed in aVL	-	-	+	-	-	-
No q allowed in I	+	+	+	-	-	-
R peak time greater than 60 ms in leads V5-V6	+	+	+	-	-	-
Normal R peak time in leads V1-V2	+	+	-	-	-	-
Discordant repolarization, but after a positive QRS the T wave can be positive	-	+	+	-	-	-
Slightly elevated ST and positive, asymmetrical T wave in V1	-	-	+	-	-	-
QS in aVR with positive T wave	-	-	+	-	-	-

WHO: World Health Organization, AHA/ACCF/HRS: American Heart Association/American College of Cardiology Foundation/Heart Rhythm Society, ESC: European Society of Cardiology, MADIT-CRT: Multicenter Automatic Defibrillator Implantation With Cardiac Resynchronization Therapy, reverse: resynchronization reverses remodeling in systolic left ventricular dysfunction.

**Table 2:** ECG signs detectable for the human eye on the conventional 12-lead ECG predicting response or non-response to CRT in different QRS morphologies.

Predictors of CRT Response	Predictors of CRT Non-Response
True LBBB	
▪ QRS duration ≥ 130 ms in women and ≥140 ms in men ▪ Mid-QRS notching and/or slurring in two contiguous leads in V1-2 or I-aVL, V5-V6 ▪ Absence of q waves in the lateral leads ▪ Absence of R wave in V1 (≥1 mm) ▪ 45 ms ≤ between the peak of the R to the nadir of the S wave in V1	
**Non-true LBBB and non-LBBB**	
▪ QRS duration ≥ 150 ms ▪ Mid-QRS notching or/and slurring in one lateral lead ▪ Masquerading bundle branch block ▪ ID in lead V6 > 60 ms ▪ QR-max index > 120 ms	▪ More than 2 notches on the R wave or the nadir of the S wave. ▪ <32.5 ms to the beginning of the QRS fragmentation from the QRS onset and a longer fractionation duration ▪ Lead one ratio < 12
**All QRS morphologies**	
▪ ID in lead I ≥ 110 ms ▪ ID in lead aVL ≥ 130 ms ▪ ID/QRS duration > 0.69 in lead I ▪ [ID in lead I − ID in lead V1] >90 ms ▪ [aVLID − aVFID]/QRSd >25% ▪ [V5ID − V1ID]/QRSd >25% ▪ Large R/S in V6 (absence of deep S wave)	▪ QRS duration < 130 ms

CRT: cardiac resynchronization therapy, LBBB: left bundle branch block, non-LBBB: non-left bundle branch block, ID: intrinsicoid deflection, QRSd: QRS duration.

## Data Availability

The data that support the findings of this study are openly available.

## Supplementary Materials

The following supporting information can be downloaded at: https://www.mdpi.com/article/10.3390/jcdd10100425/s1, Table S1: Sensitivity and specificity values for ECG signs associated with CRT response from the original publications (if they were available).
