# Supplementary material for: Useful Electrocardiographic Signs to Support the Prediction of Favorable Response to Cardiac Resynchronization Therapy
Source: J Cardiovasc Dev Dis. 2023 Oct 14;10(10):425. doi: 10.3390/jcdd10100425 (PMC10607456; doi:10.3390/jcdd10100425)
Supplement: Supplementary file 1 [file jcdd-10-00425-s001.zip › jcdd-2576336-supplementary.pdf]

Review

Useful electrocardiographic signs helping the prediction of favorable response to cardiac resynchronization therapy

Supplementary material

Supplementary Table S1. Sensitivity and specificity values for ECG signs associated with CRT response from the original publications (if they were available).

Predictors of response	Sensitivity	Specificity
**True-LBBB**		
QRS duration ≥ 130 ms in women and ≥ 140 ms in men<br>Mid-QRS notching and/or slurring in two contiguous leads in V1-2 or I-aVL, V5-V6	71.3 – 40%	40.0 – 64.1%
Absence of q waves in the lateral leads	21%	87%
Absence of R wave in V1 (≥1 mm)	67.6%	53.9%
45 ms ≤ between the peak of the R to the nadir of the S wave in V1	86.1%	66.7%
**Non-true LBBB and non-LBBB**		
ID in lead I ≥ 110 ms, ID in lead aVL ≥ 130 ms and ID/QRS duration > 0.69 in lead I	83.0%	81.0%
Masquerading bundle branch block	81.0%	71.0%
ID in lead V6 > 60 ms	82.8%	78.8%
QR-max index > 120ms	63.4 – 91.9%	82.8 – 56.7%
[ID in lead I – ID in lead V1] >90 ms<br>[aVLID – aVFID]/QRSd >25%<br>V_5ID – V_1ID]/QRSd >25%	95.7 – 100%	14.3 – 50%
**Predictors of non-response**	**Sensitivity**	**Specificity**
**Non-true LBBB and non-LBBB**		
More than 2 notches on the R wave or the nadir of the S wave	68.9%	81.6%
< 32.5 ms to the beginning of the QRS fragmentation from the QRS onset and a longer fractionation duration	83.3%	85.7%
Lead one ratio <12	33.3%	77.1%

LBBB: left bundle branch block; ID: intrinsicoid deflection.
